# MarrowCellDLD: a microfluidic method for label-free retrieval of fragile bone marrow-derived cells

**DOI:** 10.1038/s41598-023-47978-w

**Published:** 2023-12-18

**Authors:** Gloria Porro, Rita Sarkis, Clara Obergozo, Lucie Godot, Francesco Amato, Magali Humbert, Olaia Naveiras, Carlotta Guiducci

**Affiliations:** 1 Laboratory of Life Sciences Electronics, École Polytechnique Fédérale de Lausanne (EPFL), Lausanne, Switzerland; 2 Laboratory of Regenerative Hematopoiesis, Université de Lausanne (UNIL), Lausanne, Switzerland; 3 Hematology Service, Departments of Oncology and Laboratory Medicine, Lausanne University Hospital (CHUV), Lausanne, Switzerland

**Keywords:** Stem-cell research, Haematopoietic stem cells, Mesenchymal stem cells, Isolation, separation and purification, Lab-on-a-chip

## Abstract

Functional bone marrow studies have focused primarily on hematopoietic progenitors, leaving limited knowledge about other fragile populations, such as bone marrow adipocytes (BMAds) and megakaryocytes. The isolation of these cells is challenging due to rupture susceptibility and large size. We introduce here a label-free cytometry microsystem, MarrowCellDLD, based on deterministic lateral displacement. MarrowCellDLD enables the isolation of large, fragile BM-derived cells based on intrinsic size properties while preserving their viability and functionality. Bone marrow adipocytes, obtained from mouse and human stromal line differentiation, as well as megakaryocytes, from primary human CD34+ hematopoietic stem and progenitor cells, were used for validation. Precise micrometer-range separation cutoffs were adapted for each cell type. Cells were sorted directly in culture media, without pre-labeling steps, and with real-time imaging for quality control. At least 10^6^ cells were retrieved intact per sorting round. Our method outperformed two FACS instruments in purity and yield, particularly for large cell size fractions. MarrowCellDLD represents a non-destructive sorting tool for large, fragile BM-derived cells, facilitating the separation of pure populations of BMAds and megakaryocytes to further investigate their physiological and pathological roles.

## Introduction

The bone marrow (BM) constitutes the primary site of hematopoiesis, where maturing hematopoietic cells and supporting stromal cells coexist within a complex microenvironment ensuring the tightly regulated production of up to 10^12^ blood cells daily^1^. However, certain BM cell types, namely bone marrow adipocytes (BMAds) and megakaryocytes (MKs), have eluded comprehensive characterizations due to their fragile nature and considerable size when fully mature, complicating their isolation via conventional methods such as flow cytometry^2^. Consequently, they are often underrepresented or absent in critical studies such as single-cell RNA sequencing-based atlases^3–6^.

Mature megakaryocytes are large cells, 50–100 μm in diameter^7^, which produce platelets, the cell fragments mediating blood clotting. Despite efforts to decode the impact of diverse MK phenotypes on platelet generation and hematopoietic progenitor fate, the lack of robust functional studies has hindered conclusive determinations^7^. While in vitro differentiation of megakaryocytes from hematopoietic stem cells offers insights into megakaryopoiesis and platelet function^8^, the isolation of high-purity, viable MK subpopulations for mechanistic investigations remains a hurdle^8–10^. The exigency of separating mature MKs from their precursors is critical to define differentiation requirements and optimize ex vivo platelet production. Although megakaryocytes express specific surface markers facilitating their separation through flow cytometry^7,11,12^, their rarity and susceptibility to shear stress forces limit their isolation. Indeed, FACS, the gold-standard method for high-throughput cell sorting, loses efficacy when applied to cells with either large size, inherent fragility, and/or high buoyancy^13–15^.

Sorting bone marrow adipocytes is even more restrictive than MKs. BMAds constitute the most frequent BM stromal cells in larger mammals^16^, varying in size from 30 to 40 μm in mice to 80–100 μm in humans^17–19^. Historically regarded as passive space fillers, BMAds are now recognized as regulators of energy storage, bone metabolism, and hematopoiesis^20–22^. As the interest in their composition, function, and heterogeneity has increased^23–26^, their difficult isolation and preservation upon sorting have hindered research progress^2^. Additionally, since fully-lipidated mature adipocytes lack specific surface markers, neutral lipid dyes are often used for their FACS-based sorting. Yet, the labeling steps are time-consuming and affect cell viability. Notably, lipid dyes are not exclusive to mature adipocytes since they also stain progenitor cell membranes and early differentiation stages^13,27–29^. FACS sorting combining lipid staining with forward scatter (FSC) and side scatter (SSC), which reflect cell size and granularity, is a common strategy^14,30–33^. Recently, a size-based FACS protocol relying solely on FSC/SSC parameters has been proposed to isolate large unilocular mature adipocytes, albeit restricted to fixed cells and requiring components often unavailable in standard instruments^13^. Given these limitations, buoyancy-based isolation protocols have emerged as an alternative to FACS for the isolation of adipocytes as heterogeneous populations of different sizes and lipid contents^18^. An alternative strategy to separate live in vitro differentiated BMAds based on size, and thus also on lipid content, would greatly complement the need for homogeneous populations. This approach could overcome the non-specificity of lipid dyes while simplifying sample preparation and preserving cell viability across all maturation stages.

Microfluidic cell separation techniques offer a promising alternative to FACS sorting. Specifically, continuous-flow microfluidic systems are operated at significantly lower pressures than flow cytometry, minimizing shear stress forces. Label-free approaches have been implemented with various microfluidic configurations, such as deterministic lateral displacement (DLD). DLD consists of a flow-through microchamber with arrayed micropillars that physically displace large particles, enabling their separation^34–37^. Notably, the distances between DLD micropillars are wider than the particles in the processed sample, avoiding compressing even the largest particles in the mixture. Previous research on DLD sorting successfully showed the separation of red and white blood cells from whole blood, achieving post-sorting viability exceeding 90%^38,39^. Other DLD studies showed the enrichment of human BM skeletal stem cells from expansion of blood extracts^40^ and isolation of circulating tumor cells from undiluted blood^41–43^, achieving throughputs of up to 10^6^ cells per second^42^. Significantly, Huang et al*.* introduced a DLD device to isolate nucleated from non-nucleated red blood cells with a low-shear pillar array obtained by maximizing the gap size, along with the channel height by deep reactive ion etching of silicon^44^. Leveraging this high volumetric capacity array design, coupled with the parallelization of 48 sorting modules on the same chip, they achieved the high-throughput separation of 10^7^ cell/s. A noteworthy commercial DLD implementation is the Curate^®^ Cell Processing System from CurateBio^45,46^, which sorts white blood cells from plasma at 400 mL/hour by running multiple separation microchambers in parallel.

Here, we introduce a novel DLD architecture, MarrowCellDLD, specifically designed to sort large, fragile cells derived from bone marrow cultures, taking advantage of the characteristic size increase that accompanies cell differentiation of BM lineages, whose fragility has greatly limited comprehensive studies. Concretely, MarrowCellDLD is a fluid dynamic DLD microsystem purifying mature BMAds and MKs from progenitor cells and early stages of differentiation, exclusively based on their size difference. Through extensive testing on in vitro derived BMAds from mouse and human, as well as primary MKs, we demonstrate effective separation without compromising viability or functionality, enabling cell culture post-sorting. We validate the phenotype of the isolated fractions and compare our method to FACS in terms of purity and functional cell yield. This microfluidic system represents the first non-destructive, phenotype-based label-free sorting method of pure fractions of mature BM adipocytes or megakaryocytes obtained from in vitro differentiation with a precisely defined size range.

## Results

### High-purity isolation of mature OP9 adipocytes

**Figure 1:**
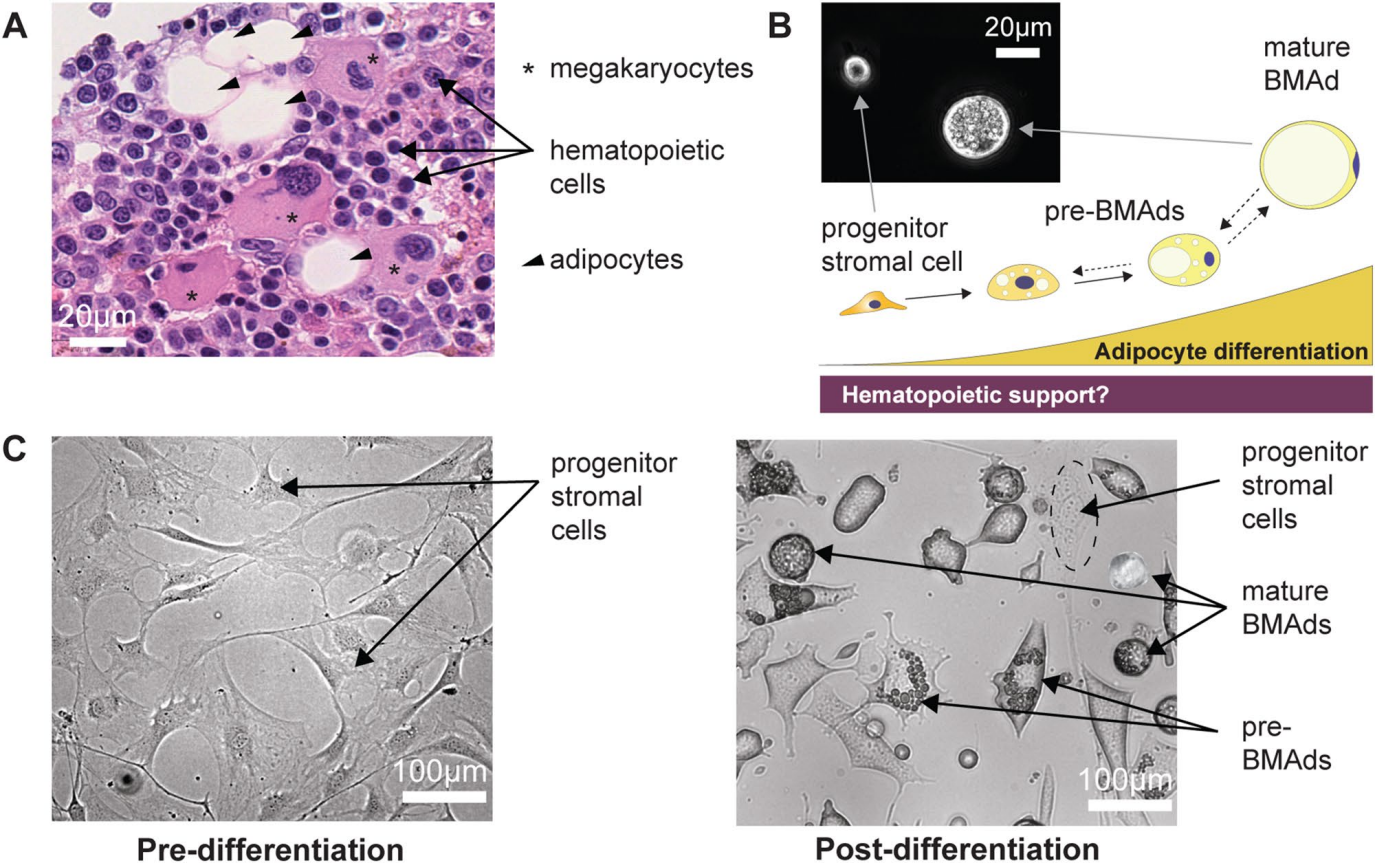
Bone Marrow niche and Heterogeneous Adipocytic Differentiation in vitro. (**A**) Hematoxylin and eosin-stained slide of human bone marrow (BM) trephine biopsy imaged at 40X magnification using a Nanozoomer S60, revealing hematopoietic cells (labeled with arrows), megakaryocytes (labeled with asterisks), and bone marrow adipocytes (BMAds) (labeled with arrow heads). (**B**) Schematic representation of the BMAd differentiation axis as a study model for the relationship between hematopoiesis and adipogenesis. Inset: phase contrast imaging of cells in suspension at the progenitor and mature BMAd stages, highlighting the size difference between these two populations. (**C**) OP9 progenitor cells seeded at 20,000 cells/cm^2^ (undifferentiated-OP9) show a homogeneous fibroblastic-like cell structure in adherent cultures. Following in vitro induced adipocytic differentiation (induced-OP9, 6 days post-differentiation), the sample contains various stages of maturation: progenitors (fibroblast morphology), pre-BMAds (cells with limited lipid droplet accumulation), and mature BMAds (round cells filled with lipids).

The bone marrow (Fig.  1A) is a complex tissue with diverse cellular species. In addition to hematopoietic cells, it includes MKs and BMAds, both characterized by their large size and fragility in suspension. To develop our sorting device, we first utilized OP9 cells, a non-clonal line of bone marrow-derived mouse stromal cells known as a robust adipogenesis model^47,48^. After in vitro adipocytic induced differentiation, OP9 progenitors accumulated lipid droplets and underwent the expected size increase (Fig. 1B, in suspension). Figure 1C shows pre- and post-differentiation OP9 cultures. The differentiation outcome depended on passage number and confluency at induction, thus induced-OP9 samples resulted in varying ratios of differentiation stages, from progenitors to mature adipocytes, ranging from 7 μm to 40 μm in size.

**Figure 2:**
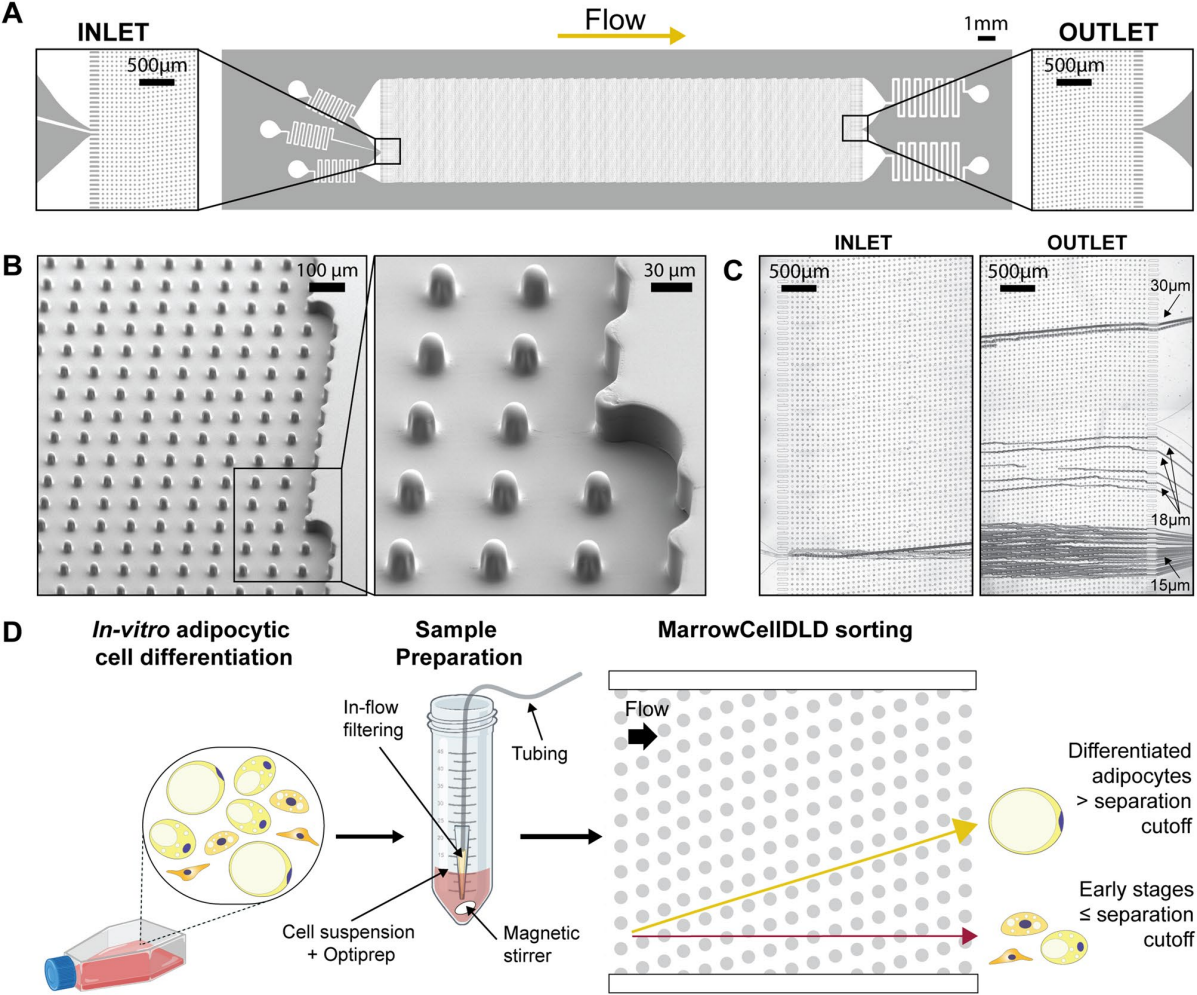
MarrowCellDLD device and operation. (**A**) MarrowCellDLD chip: insights on inlet and outlet regions terminated with 10 rows of straight pillars respectively before and after the MarrowCellDLD array active region (tilted array). (**B**) Scanning electron microscopy images of the MarrowCellDLD array chip with a 19 μm separation cutoff (critical size). (**C**) Inlet and outlet trajectories of polystyrene microbeads of 15, 18, and 20 μm in size transiting a MarrowCellDLD chip with 19 μm critical size. (**D**) Experimental workflow to sort differentiated adipocytes by MarrowCellDLD. After adipocytic differentiation *in vitro*, the induced-OP9 sample contains a mixture of progenitor cells, early stages of differentiation, and mature adipocytes. After trypsinization, the cellular sample is suspended in its original culture media supplemented with Optiprep, then placed at the inlet reservoir connected to the tubing responsible for injecting the sample into the MarrowCellDLD device. A custom in-flow filtering system embedded in the tubing ensures the injection of a single-cell suspension, and the sample is continuously stirred to achieve homogeneity. Within the sorting module, mature adipocytes larger than the critical size for separation should be forced to follow the array angle (displacement mode), allowing for their isolation. Conversely, early stages of differentiation and progenitors should move parallel to the flow (zig–zag mode). The two fractions are thus predicted to be physically separated and can be collected at distinct outlets.

**Figure 3:**
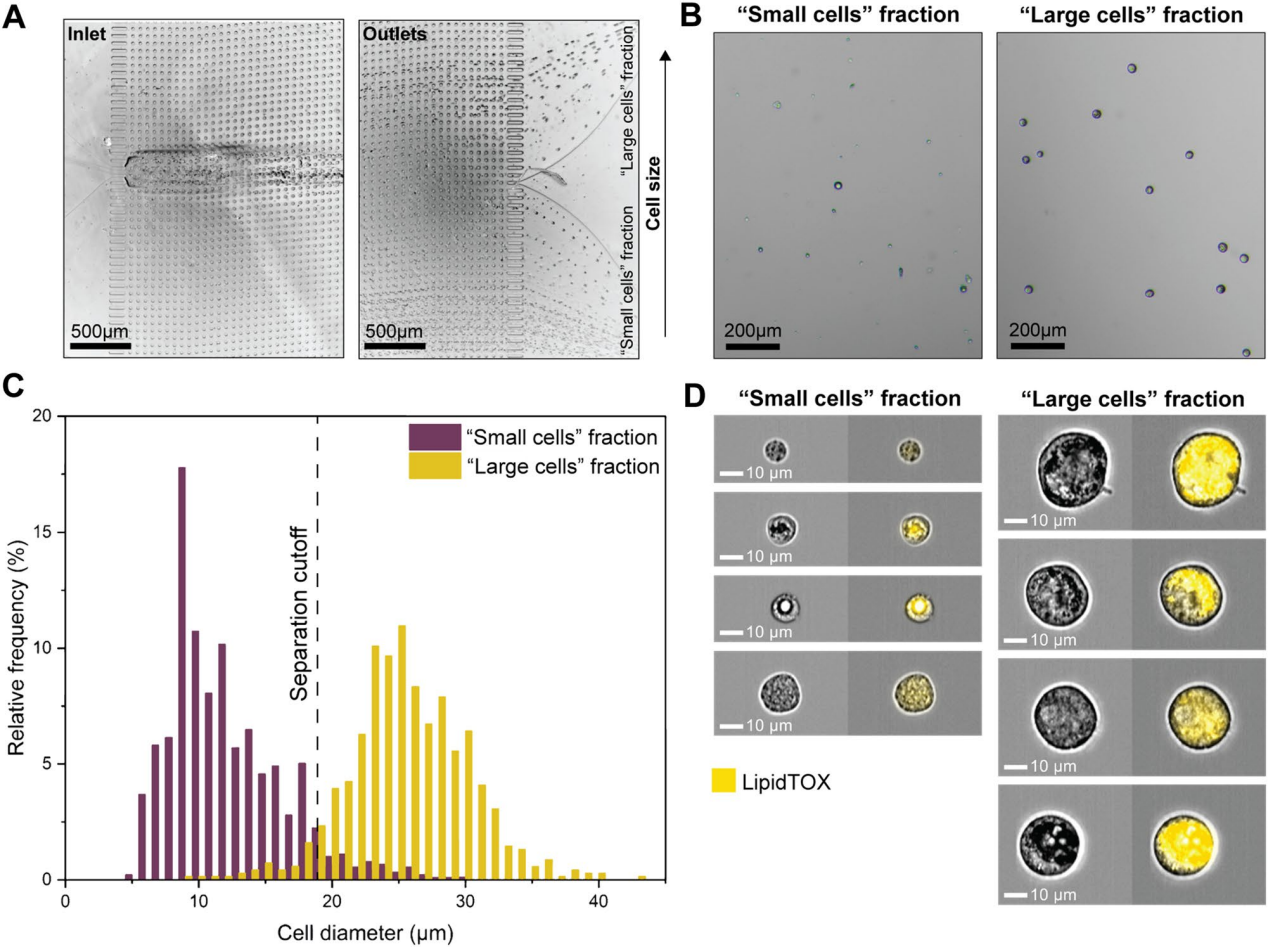
Fluid Dynamic MarrowCellDLD Sorting of Induced-OP9 Cells. (**A**) Trajectories of cells entering (inlet) and exiting (outlets) the microfluidic system. The test involved injecting induced-OP9 cells after 6 days of differentiation at a concentration of 500,000 cells/mL. (**B**) Phase contrast microscopy of outlet fractions after MarrowCellDLD sorting an induced-OP9 mixture. (**C**) Cell diameter distributions of outlet fractions (« small cells » fraction, violet; « large cells » fraction, yellow) after sorting induced-OP9s by MarrowCellDLD with a 19 µm separation cutoff. Phase contrast microscopy of cells collected at the outlets and replated after sorting as in (**B**) enabled cell size quantification in QuPath0.3.2. Data are displayed as relative frequencies over 150 cells per fraction from n = 4 sorting experiments. (**D**) ImageStream flow cytometry imaging (brightfield and LipidTOX-stained) of cells from the two fractions separated by MarrowCellDLD. MarrowCellDLD sortings were performed with a 19 µm separation cutoff and applied pressure of 20 mbar.

The microchip layout and SEM images of the sorting module are presented in Fig. 2A and 2B, and supplementary videos (SV1–2) show the microchip operation. Figure 2C and 2D depict the behavior of microbeads within MarrowCellDLD and the sorting workflow for adipocytic cultures. Figure 3A shows a sample of OP9-derived adipocytic culture (induced-OP9^48^), transiting the MarrowCellDLD sorter at the inlet and outlet. Specifically, a single-cell suspension was continuously injected and focused using lateral sheath flows (Fig. 3A, inlet). Stirring the sample prevented bias due to cell buoyancy and an embedded filtering system at the inlet tube reduced doublets and clusters (Fig. 2D). Cluster removal was essential to guarantee cell purity and continuous sample processing for up to 3–4 h without clogging issues.

The MarrowCellDLD sorting array was designed to retrieve two fractions based on a separation cutoff size determined by the array periodicity and gap between pillars. The injected induced-OP9 single-cell suspension comprised a continuous distribution of cell sizes. As expected from the design, cells exited the array spreading across the width of the main channel, which subdivides downstream into two subchannels denominated « small cells » and « large cells » fractions (Fig. 3A, outlets). Notably, the « small cells » outlet collected more events per unit time, including small debris and contaminations, whereas the « large cells » outlet was highly purified (Fig. 3B).

We tested MarrowCellDLD sorting arrays with different separation cutoffs (DLD critical sizes). The nominal separation cutoff was calculated based on the general DLD model^38^. We initially designed devices at nominal critical sizes 15, 17.5, 20, 22.5, and 25 μm and fabricated them as PDMS replicas. The gaps between the pillars in the fabricated DLD arrays measured by surface profilometry were larger than the designed ones, therefore the actual separation cutoffs resulted larger than the nominal critical sizes. We first characterized the sorting modules by processing polystyrene microbeads of different sizes (Fig. 2C). Subsequently, we tested the five different array geometries with induced-OP9 samples and observed the sorted fractions by phase contrast microscopy. Nominal critical sizes of 22.5 μm and 25 μm were too large for retrieving mature OP9 adipocytes. We found that a nominal critical size of 15 μm corresponded to a 19 μm separation cutoff, which was ideal for isolating mature OP9 BMAds (Fig. 3C). The gap of 42 ± 1 μm in this array was sufficient to preserve intact mature adipocytes with minimal blockage (few units per sorting run).

Specifically, Fig. 3C shows the size distributions of the two output fractions after sorting induced-OP9s by MarrowCellDLD with a 19 µm separation cutoff. Cells collected at the outlet reservoirs were imaged by phase contrast microscopy, and QuPath0.3.2 software^49^ was used for cell diameter quantification (n = 4 independent experiments with 150 cells per fraction). We found that 97 ± 2% of cells within the « large cells » fraction were above the 19 μm cutoff, representing an approximately three-fold enrichment compared to the original induced-OP9 adipocytic sample (with only 38% above 19 μm). Consistently, the « small cells » fraction contained 96% ± 3% of cells below the separation cutoff. Independent sorting experiments through different MarrowCellDLD devices showed reproducible purity for both fractions, independent from the original mixture composition. The combined size distribution of the sorted fractions matches that of the original sample, indicating the preservation of cell size dynamics. Additionally, we further characterized devices with a nominal critical size of 20 μm, as they could provide the finest separation among the ones tested in our study. Figure S1 shows the sorting outcome for a device with a nominal critical size of 20 μm that corresponded to an actual separation cutoff of 24 μm, achieving 90–93% purity over the « large cells » fraction for two independent sorting experiments. It is worth noting that with both geometries we could reliably retrieve intact BMAds above 35 μm in diameter, the size of the largest adipocytes found in the murine BM, as defined within the intact tissue.

To validate our findings, we observed the populations sorted by MarrowCellDLD by ImageStream flow cytometry imaging (Fig. 3D). LipidTOX, a lipophilic stain commonly used for adipocyte FACS sorting, was used to tag the lipid-laden cells and LipidTOX signals quantified by Imagestream analysis of the sorted fractions (Fig. S2). Indeed, all cells within the « large cells » fraction exhibited positive LipidTOX staining (98% ± 2%, n = 3), indicating significant lipid accumulation typical of mature BMAds. Large adipocytes and diverse levels of lipid drop coalescence were observed. Interestingly, several cells in the « small cells » fraction (63% ± 12%, n = 3) also displayed LipidTOX positivity at varying intensities. Some pre-BMAds showed small lipid droplets coalescing, producing a significant LipidTOX signal even if not fully mature. These results suggest that BMAds sorting based solely on lipid staining intensity may not be sufficient to differentiate between adipocyte maturation stages. Gently purifying adipocyte populations with a precisely defined size range can help to overcome this limitation.

### Phenotype of sorted induced-OP9 adipocyte fractions

In addition to ImageStream flow cytometry imaging, BODIPY staining and phase contrast microscopy were used to interrogate the phenotype and neutral lipid content of the cells in the small and large cell fractions as compared to the unsorted mix (Fig. S3A). To confirm the presence of mature BMAds in the « large cells » fraction and morphologically discern BMAds from their progenitors or intermediately differentiated cells, we employed a BODIPY fluorescence stain, indicative of cellular lipidic content and thus of the extent of adipocyte maturation upon differentiation. Compared to the unsorted mixture comprising 81% BODIPY-positive cells, we found the great majority (95%) of cells in the « large cells » sorted fraction to stain positive for BODIPY (Fig. S3C-D). Conversely, a small fraction (18%) of cells within the « small cells » fraction was stained for BODIPY, confirming the enrichment of smaller, non-lipidated cells (Fig. S3B). Overall, we concluded that MarrowCellDLD sorting of induced-OP9 adipocytic cell suspensions could successfully separate large, lipidated, intact BMAds at high purity from unlipidated or adipogenesis-refractory precursors as determined by the phenotype and lipid content of the sorted cells.

### Viability and functionality of sorted induced-OP9 adipocytes

**Figure 4:**
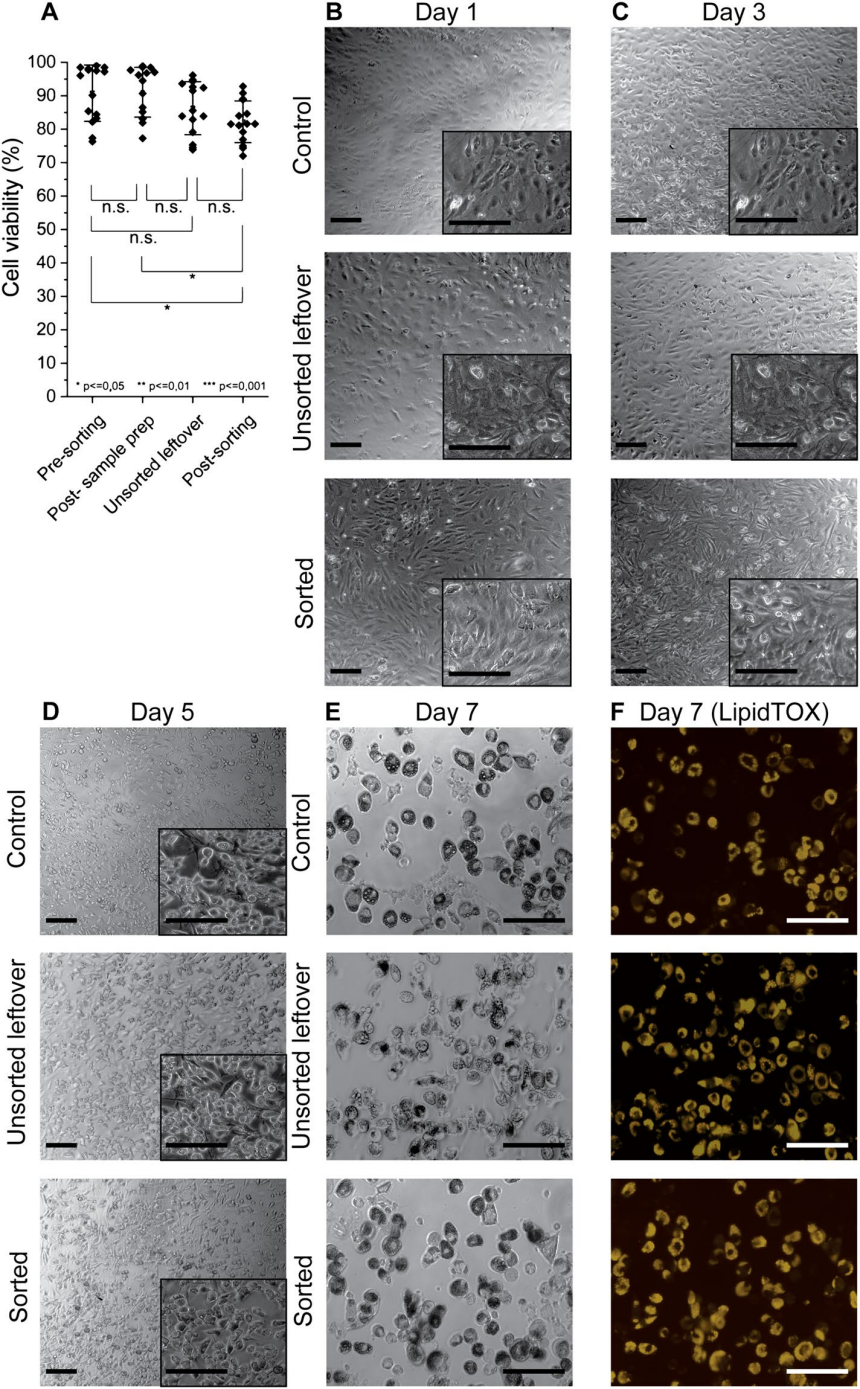
Cell viability and functionality of OP9 cells after MarrowCellDLD sorting. (**A**) Cell Viability measurements: (i) pre-sorting, (ii) post-sample preparation, (iii) unsorted leftover, and (iv) cells collected at outlets post-sorting (small and large cell fractions). Statistical significance was evaluated by the Student’s t-test for independent samples (n = 15 MarrowCellDLD sorting experiments on induced-OP9 samples). (**B**–**F**) Time-sequential images at different days post-plating following MarrowCellDLD sorting of undifferentiated OP9 cells. Phase contrast and fluorescence (Yellow, LipidTOX staining) images for control cells (unsorted), unsorted leftover (unsorted sample residual at the inlet reservoir after 3 h sorting), and sorted (collected at the «small cells » fraction outlet) after induced adipocytic differentiation for 6 days. Scale bar: 100 µm. MarrowCellDLD sortings were performed with a 19 µm separation cutoff and applied pressure of 20 mbar.

Next, we evaluated the viability of induced-OP9s before and after MarrowCellDLD sorting (n = 15, with approx. 100 cells per experiment) (Fig. 4A). Cell viability was measured using Trypan Blue and a hemocytometer. Sample filtering and Optiprep addition did not significantly influence cell viability (pre-sorting viability: 91% ± 7%; post-sample preparation viability: 91% ± 8%). Cells remaining in the input vial after the sorting process exhibited comparable viability (unsorted leftover viability: 86% ± 8%). As for the sorted cells, a significant but modest decrease in viability (82% ± 6%) was observed as compared to the pre-sorting sample, but the difference was no longer significant when compared to the unsorted leftover. Overall, viability never fell below 70% for all sorting experiments performed.

As shown in Fig. 4B–F, we further assessed post-sorting viability and functionality by comparing (i) undifferentiated OP9 cells that were subject to neither sample preparation nor sorting (Control), (ii) undifferentiated-OP9 cells remaining unsorted at the input vial (Unsorted leftover), and (iii) sorted undifferentiated OP9 progenitor cells (Sorted). Undifferentiated cultures were chosen for these experiments to ensure adherence for downstream differentiation assays, as otherwise, the low adherence displayed by mature adipocytes from induced-OP9 cultures would have made the sorted cell fractions very difficult to compare. Time-lapse phase contrast microscopy and fluorescence (LipidTOX) images were captured on days 1, 3, 5, and 7. Notably, the sorted OP9 cells, unsorted leftover cells, and control cell fractions all adhered and proliferated at days 1 and 3, which further confirms post-sorting viability. To determine functionality, we induced differentiation of the three samples. Subsequent imaging on days 5 and 7 revealed clear evidence of adipocytic differentiation, confirmed by LipidTOX staining, which reflected lipid droplet accumulation. Therefore, neither the sorting nor the sample preparation and stirring affected the ability of OP9 progenitors to differentiate in BMAds. Overall, we could thus validate the reliable performance of MarrowCellDLD in isolating different phenotypes of induced-OP9 adipocytic cells with preserved viability and functionality post-sorting.

### Comparison with FACS sorting

We compared the performance of MarrowCellDLD with FACS sorting, the gold-standard approach for precise cell sorting at high throughput. FACS sorting relied on LipidTOX intensity to isolate adipocytic cultures into three distinct populations after gating on single viable cells (PI-negative or DAPI-negative), classified respectively as Low, Medium, and High LipidTOX fractions. Note that the FACS-sorting gates for the High LipidTOX fractions were inclusive of all detectable FSC/SSC high events. This gating approach (Fig. S4A-B) was implemented employing two different FACS instruments: BD FACSAria™ III (BD Biosciences) at a nozzle pressure of 20 psi and MoFlo Astrios EQ (Beckman Coulter) at a reduced pressure of 10 psi. Lower pressure is expected to preserve fragile adipocytes but implies a slower sorting process.

**Figure 5:**
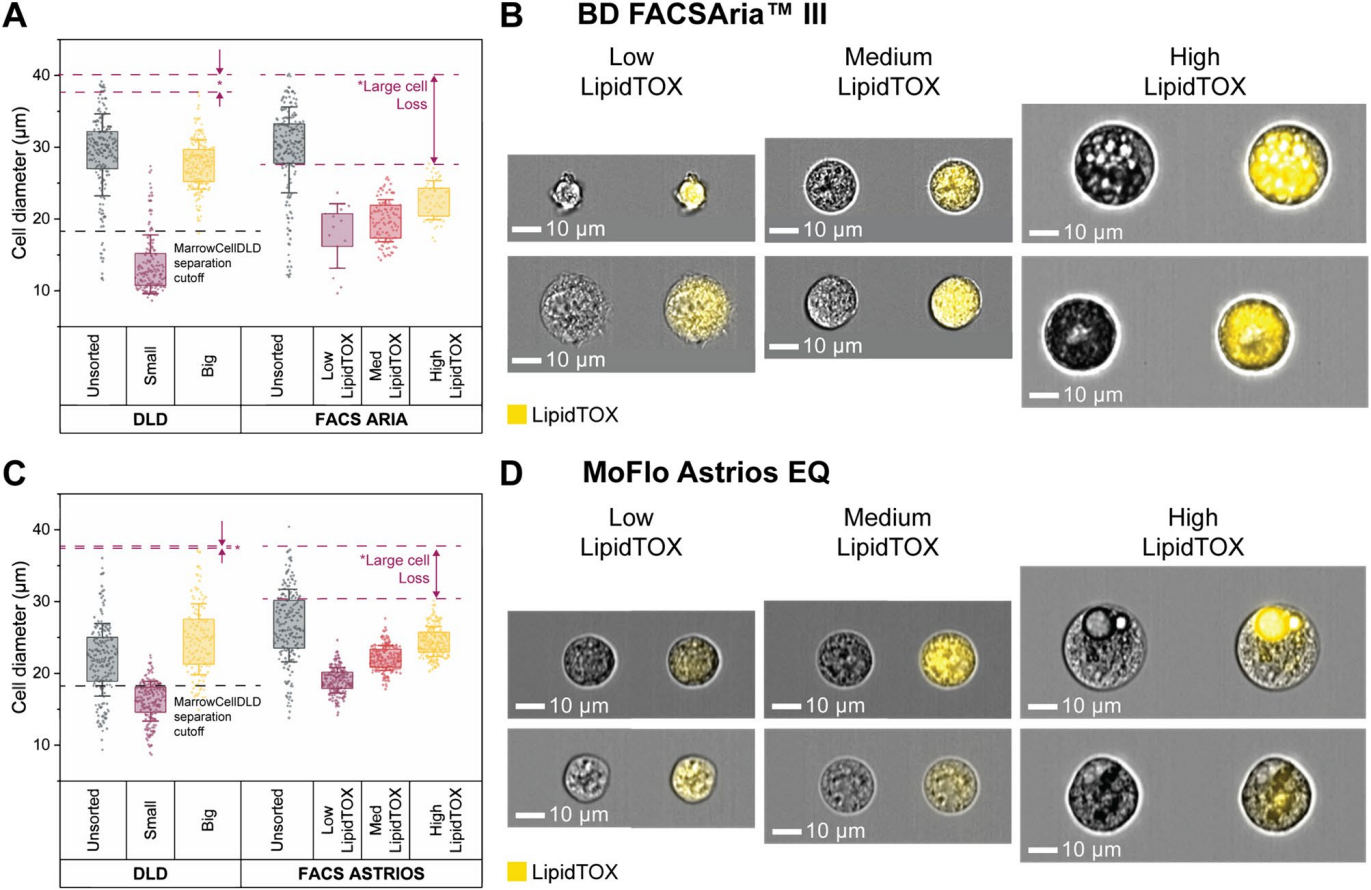
Comparison between MarrowCellDLD and FACS sorters BD FACSAria™ III and MoFlo Astrios EQ. MarrowCellDLD size-based separation is compared to FACS sorting based on LipidTOX staining for lipidic content. (**A**, **C**) Post MarrowCellDLD or FACS sorting, cells from the unsorted sample and the sorted fractions were imaged by ImageStream flow cytometry. Distributions of sizes of single viable cells of each fraction after sorting by MarrowCellDLD, FACS Aria, and MoFlow Astrios FACS instruments. Cell diameters are extracted by ImageStream analysis for each method (200 cells per group or total cells analyzed in the fraction plotted from a single experiment, selected from n = 2 biological replicates using BD FACSAria™ III cell sorter and n = 3 biological replicates using MoFlo Astrios EQ cell sorter). Data are displayed with standard deviation as error bar, and box plot representing the 25% to 75% data points of the total sample. (**B**, **D**) Representative ImageStream images of brightfield and LipidTOX-stained cells from the FACS sorted fractions obtained by FACS Aria and MoFlo Astrios, respectively. MarrowCellDLD sortings were performed with a 19 µm separation cutoff and applied pressure of 20 mbar.

Following FACS sorting, all output fractions were analyzed by ImageStream to extract the diameter of single viable cells (Fig. S4C). Figure 5 shows the sorting outcomes with Aria and Astrios FACS instruments, with cell size distributions in Fig. 5A and 5C, respectively. Representative ImageStream micrographs of the MarrowCellDLD fractions are shown in Fig. 5B and 5D, as compared to Fig. 3C. A single FACS sorting experiment is directly compared with a single MarrowCellDLD sorting of induced-OP9s with the same passage number, to compare samples with similar size dynamics. The unsorted cells were subjected to the same sample preparation protocols as the sorted fractions and kept at the input reservoirs for the whole process for both MarrowCellDLD and FACS sortings.

For the FACS Aria versus MarrowCellDLD comparison, unsorted cells were highly differentiated, with 75% cells above 26 μm (Fig. 5A and 5B). MarrowCellDLD effectively isolated cells above the predetermined 19 μm cutoff, purifying mature adipocytes and even preserving cells larger than 35 μm. Conversely, the high LipidTOX fraction obtained after FACS Aria sorting surprisingly lacked cells above 30 μm. Notably, we did not observe any intact unilocular adipocytes after sorting by FACS Aria. Despite ImageStream confirming the higher degree of lipid accumulation within the high LipidTOX fraction, cells in the other two sorted populations were to a lesser extent positive for the lipophilic stain and overlapped in terms of size. We thus concluded that LipidTOX gating alone was insufficient to discriminate between different degrees of lipidation and that the vast majority of large mature adipocytes was lost during the FACS Aria sorting process.

We then moved to compare the MarrowCellDLD device to the low-sorting-pressure MoFlo Astrios FACS instrument. We included FSC/SSC gating into the FACS-sorting strategy to better discriminate the Low and Medium LipidTOX populations^13,47,50^. For this set of experiments, the unsorted induced-OP9 samples in the MarrowCellDLD experiment exhibited a narrower range of cell sizes (Fig. 5C), therefore including a lower proportion of large, mature adipocytes than for Fig. 5A. As shown in Fig. 5C, upon ImageStream analysis we found that MarrowCellDLD isolated cells above the 19 μm separation cutoff and preserved cells larger than 35 μm. For the MoFlow Astrios FACS-sorted fractions, we observed LipidTOX-positive cells in all three fractions, and a harmonious increase in size proportional to the lipid signal (Fig. 5D). The MoFlo Astrios sorter could retrieve more intact viable high-LipidTOX cells than the FACS Aria instrument, but again produced losses of large high-LipidTOX cells (Fig. 5C). Specifically, cells within the MoFlo Astrios high-LipidTOX fraction displayed lipid droplet accumulation but often did not exhibit complete differentiation, and unilocular adipocytes were not observed (Fig. 5D). We, therefore, concluded that contrary to the MarrowCellDLD device, and although less damaging, the low-pressure MoFlow Astrios FACS sorting still lacked the gentleness required to preserve all fragile adipocytes.

Then, we compared the MarrowCellDLD performance to the MoFlow Astrios FACS instrument in terms of processing time and yield (Table 1). While MarrowCellDLD is label-free, FACS requires LipidTOX staining incubation (30 min) and washing (15 min) steps before sorting. Additionally, centrifugation for medium exchange to a FACS buffer potentially harms the cells and introduces buoyancy-based biases. For a sample of 1.5 × 10^6^ cells, i.e. the sample size of induced-OP9 cells in a T25 culture flask, MarrowCellDLD required 180 min of sorting time, while FACS Astrios approximately 120 min. Including in the total processing time the sample preparation times, which for FACS include staining incubation times, the FACS process required 170 min in total and MarrowCellDLD took approximately 185 min, both within the same order of magnitude.

**Table 1:** Performance comparison. Considering a sample mixture of 1.5 × 10^6^ induced-OP9 cells in 3 mL (T25 cell culture flask post-differentiation) entirely processed by MarrowCellDLD or FACS Astrios to isolate mature adipocytes, the table summarizes for each sorting method (i) the sample preparation time, (ii) the sorting time, (iii) the total processing time (sample preparation and sorting), (iv) the percentage of large cells lost in the process compared to the unsorted sample calculated as the percentage of unsorted cells above the mean + standard deviation in the high LipidTOX fraction, and (v) the dilution introduced by the sorting process. MarrowCellDLD sortings were performed with a 19 µm separation cutoff and applied pressure of 20 mbar.

	MarrowCellDLD	FACS
Sample preparation time	5 min (trypsin + add Optiprep + filter)	50 min (trypsin + stain LipidTOX/DAPI, wash, filter)
Sorting time	180 min (full sample)	120 min (full sample)
Total processing time	185 min (full sample)	170 min (full sample)
Large cell loss	7%	50%
Dilution	40X	No dilution (FACS yield 10%)

To estimate losses of large adipocytes, we compared the size distributions of sorted and unsorted cells (Fig. 5C). MoFlow Astrios FACS sorting could not recover 50% of cells larger than 19 μm in the original sample, calculated as the percentage of unsorted cells above the mean + standard deviation in the high LipidTOX fraction. In contrast, for MarrowCellDLD, the comparison between the large cell fraction and the unsorted population revealed that only 7% of the large cells were lost during sorting. Finally, we should note that FACS does not entail a dilution of the original sample, while MarrowCellDLD, at this stage of our design, introduces a 40-fold dilution.

Finally, we tested the effectiveness of isolating large lipidated cells from the induced-OP9 single-cell suspensions using the floatation-based protocol developed to isolate primary adipocytes from femoral surgical debris, introduced by Attané et al.^18^. Unfortunately, and as reported by the authors (personal communication) we were not able to obtain a floating layer of high-buoyancy adipocytes from our in vitro differentiated murine stromal cultures even after long waiting times post-centrifugation (Supplement Fig. S5), as opposed to the primary human BMAds their technique was developed for. The expected cell diameter heterogeneity of induced-OP9 adipogenic cultures was retrieved within the pelleted fraction, including numerous cells larger than 35 μm. Therefore, although we could not compare the buoyancy method for mature adipocyte isolation, we could demonstrate that centrifugation enables the concentration of mature OP9-derived adipocytes to counteract the dilution introduced by MarrowCellDLD.

### MarrowCellDLD sorting of spontaneous OP9, induced MSOD, and induced megakaryocytes

**Figure 6:**
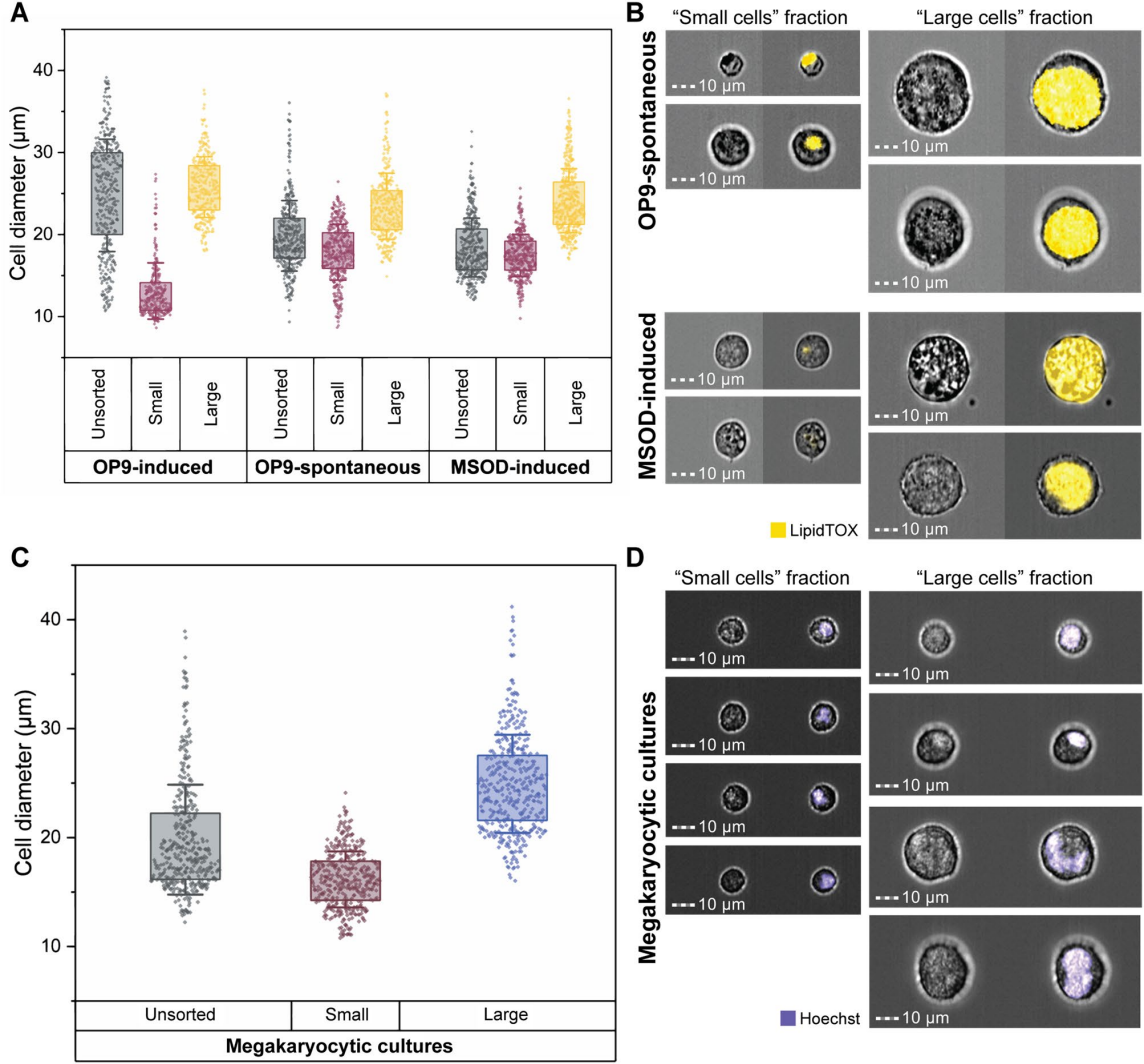
MarrowCellDLD sorting of different BM cell types. We compared the MarrowCellDLD sorting of adipocytes from (i) induced (OP9-induced) or (ii) spontaneously differentiated (OP9-spontaneous) mouse-derived OP9 stromal cells, as well as (iii) human-derived MSOD progenitors (MSOD-induced). (**A**) After MarrowCellDLD sorting, cells were stained by DAPI and imaged by ImageStream flow cytometry to quantify the diameter of DAPI-negative single cells (n = 2 experiments for each cell type, with 400 cells per group). (**B**) Representative ImageStream micrographs of OP9-spontaneous and MSOD-induced cells (brightfield and LipidTOX-stained) separated by MarrowCellDLD sorting onto « small cells » (left panels) and « large cells » (right panels) and outlet based on a predefined critical size cutoff of 19 μm. (**C**) MarrowCellDLD sorting of human megakaryocytes derived from primary CD34+ hematopoietic progenitor cells. Cells were stained by Hoechst and imaged by ImageStream flow cytometry, to quantify the diameter of the single Hoescht-positive cells (n = 2 experiments, with 400 cells per group). (**D**) Representative ImageStream micrographs upon MarrowCellDLD sorting of megakaryocytic cultures, showing megakaryocytes on the « large cells » fraction (brightfield and Hoechst-stained after MarrowCellDLD sorting). MarrowCellDLD sortings were performed with a 19 µm separation cutoff and applied pressure of 20 mbar.

MarrowCellDLD consistently demonstrated high-performance sorting of induced-OP9 adipocytes. To further validate the versatility of our device for fragile cell types, we conducted MarrowCellDLD sorting experiments on two additional adipogenesis models: spontaneously differentiated OP9 cells, where mature adipocytes are rare (Fig. 6A) and adipogenesis-induced MSOD human-derived stromal cells (Fig. 6B), as well as human megakaryocytes derived from primary CD34+ cells (Fig. 6C). For the adipocyte models, we followed the same sample preparation protocol and sorted an equal number of cells (1.5 × 10^6^ cells) in each scenario.

As expected, the post-differentiation single cell-mixture of spontaneous-OP9s, or induced-MSODs, consistently included fewer mature BMAds as compared to the previously tested induced-OP9 samples. However, even if both OP9- and MSOD-derived mature BMAds were rare in these conditions, the MarrowCellDLD device successfully separated at high purity large cells above the 19 μm separation cutoff (Fig. 6A). Specifically, spontaneous-OP9 adipocytes were collected at 90% purity and induced-MSOD adipocytes at 94% purity. ImageStream assessment of the LipidTOX-labeled fractions confirmed that cells within the « small cells » fraction corresponded to the phenotypes of unlipidated progenitor cells or early-stage lipidated cells, while fully-lipidated cells with mature BMAd phenotype were collected within the « large cells » fraction (Fig. 6B). This correlation was evident from both the intensity and area of the LipidTOX stain, as well as the cell size inferred from the brightfield images. Thus, we can conclude that MarrowCellDLD could be also used for high-purity sorting of rare BMAds, in vitro-derived from both mouse and human progenitor cells. Notably, the largest adipocytes (> 35 μm) were preserved even if extremely rare.

Finally, we tested our method to isolate differentiated megakaryocytes from primary human progenitors. Differentiation of human CD34+ hematopoietic stem and progenitor cells isolated from peripheral blood was induced in vitro. Upon megakaryocytic differentiation, progenitors undergo endomitosis resulting in large, polyploid cells, expressing the characteristic surface markers CD41 and CD42b^9^. The extent of differentiation is largely donor-dependent. Similar to OP9 and MSOD cell lines, the post-differentiation sample contained a mixture of progenitors and various stages of differentiation. Large cells within this mixture, as defined by FSC on flow cytometry and by size assessment (diameter > 19 µm) by ImageStream, were double positive for the megakaryocyte-specific markers CD41 and CD42b (Supplement. Fig. S6). We tested the capacity of the MarrowCellDLD device (19 µm separation cutoff) to sort hematopoietic cell mixtures after megakaryocytic differentiation (Fig. 6C). Cells above the separation cutoff were isolated at 96% purity and retrieved intact, including cells larger than 35 µm in diameter. Congruently, we found higher polyploidy, as measured by DNA dye Hoechst 33,258, in the « large cells » fraction as compared to the « small cells » fraction of the respective MarrowCellDLD outlets (Fig. 6D). Cell viability was not significantly affected, neither by maintaining the sample under agitation during the process nor by the MarrowCellDLD sorting procedure itself (Supplement. Fig. S7). Overall, our results confirm the robustness and adaptability of MarrowCellDLD as a powerful sorting method for various fragile cell types, based on a predefined size cutoff, including terminally differentiated cells from human sources.

## Discussion

Our study introduces MarrowCellDLD (Fig. 2), a size-based microfluidic cell sorting device to isolate BM-derived large, fragile cells including adipocytes and megakaryocytes. This method offers high-throughput sorting with low mechanical stress, through a gentle continuous-flow system operated at significantly lower pressures than traditional flow cytometry. Real-time imaging capabilities enable inherent quality control during sorting, specially valuable for cell biology research. In particular this strategy could be of interestin studying bone marrow adiposity, where assessing BMAd purity remains challenging for standardization and comparability between different studies^2^.

To validate the efficiency of MarrowCellDLD, we first defined the optimal cutoff size to obtain BMAds, and then isolated at high purity OP9-derived BMAds obtained from in vitro culture in adipogenic conditions. Adipocytic induced-OP9 samples comprised a mixture of differentiation stages with varying proportions of mature, lipidated cells. MarrowCellDLD consistently isolated intact cells above the 19 μm separation cutoff within the « large cells » fraction at 97% purity, regardless of the original composition (Fig. 3C). A separation cutoff of 24 μm was efficient for finer isolation of the largest cells in the mixture.

Microscopy and ImageStream flow cytometric imaging confirmed that all species within the MarrowCellDLD « large cells » fraction indeed showed lipid accumulation. However, when comparing the two fractions based on LipidTOX signal by ImageStream, we observed that even the small progenitor cells were positively stained, despite the large adipocytes showing a brighter signal and the presence of large coalesced lipid droplets. These observations indicate that BMAd cell line sorting methods relying solely on neutral lipid dyes^27–29^ lack specificity towards mature adipocytes, due to concurrent staining of cell membranes and to the impact of lipid droplet coalescence on fluorescence intensity, as previously suggested by Hagberg et al*.*^13^*.* While LipidTOX analysis proved valuable in characterizing the phenotype of the sorted fractions, we corroborated these findings, namely that the difference in fluorescence signal from the neutral lipid dye is inadequate for precise and consistent discrimination between mature adipocytes and stromal progenitors. Additionally, while their method demonstrated efficient size-based FACS isolation of large unilocular adipocytes, it requires a larger nozzle, reduced pressure, and additional filters, which are often unavailable for standard FACS instruments. Most importantly, despite the adapted shear stress conditions, this process cannot retrieve unfixed adipocytes.

In contrast, our size-based sorting approach offers a non-destructive process preserving within the combined fractions the original cell size distribution in the mixture, thus enabling the recovery of intact cells for analysis and culture post-sorting. The viability of induced-OP9 fractions after MarrowCellDLD sorting remained on average consistently above 80% (Fig. 4A). Previous studies on DLD devices reported even higher (> 95%) DLD post-sorting viability for skeletal stem cells^40^ or CTCs^42^. Mature BMAds are, however, notoriously more fragile. Moreover, we observed a comparable slight reduction of cell viability on leftover unprocessed samples after a few hours of experimental time, indicating that the sorting method itself is purely non-destructive, while adipocyte viability is dependent on the processing time. Mechanical stress from DLD sorting did not affect cell functionality, as progenitor OP9 cells were able to proliferate post-sorting and differentiate into adipocytes (Fig. 4B–F). The simple sample preparation, the stirring procedure, and the sample conditions during sorting could be further optimized to improve viability if pertinent. Nonetheless, MarrowCellDLD as tested already enables the retrieval and replating of very large OP9 BMAds post-sorting, sufficient for further functional studies such as co-culture of BMAd populations with a precisely defined size range, for example, in combination with defined hematopoietic progenitor populations.

We compared our method’s performance to two different FACS instruments, BD FACSAria™ III and MoFlo Astrios EQ (Fig. 5). The first operates at 20 psi 100 μm nozzle pressure and the second at 10 psi with the same diameter. Coherently, in our experiments, FACSAria preserved fewer large BMAds than MoFlow Astrios. However, even the latter instrument introduced losses as high as 50% for the larger cell fraction. The protocol introduced by Hagberg et al.^13^ suggested the use of a larger nozzle (150 μm) at 6 psi, however, these settings were not available for our setups, as is expected for most machines in research labs. MarrowCellDLD, on the other hand, preserved even the large adipocytes and separated the original mixture into two distinct subpopulations with high reproducibility. From a future perspective, we could potentially sort more than two fractions using the same approach with minimal design adaptation. The present MarrowCellDLD setup comprehends very little instrumentation and easy implementation within a cell and experimental research laboratory. It requires a three-channel pressure pump to manually adjust the inlet pressures and can be readily combined with any microscope used in research labs for cell imaging.

Mature BMAds from tissue biopsies are usually separated by floatation due to their difference in buoyancy^18^. Yet we could not isolate OP9-derived BMAds by this method. We envision that floatation could be optimized for bulk enrichment of primary BMAds, but not to precisely isolate a pure population of mature BMAds based on size. In our hands, gentle centrifugation enabled to concentrate all differentiation stages in the pellet, which was useful to compensate for the dilution introduced by the MarrowCellDLD process. At the present stage, our device introduces a 40X dilution introduced by the design of two sheath flows to focus the cellular sample stream. This dilution factor could be reduced by minimizing the volumetric capacity of the sheath flows, or introducing a module for focusing upstream of the sorting area thus eliminating the need for sheath flows^51,52^. Our protocol did not require any buffer exchange as the cells were sorted in their original culture media and could be directly replated post-sorting.

Further to the validation of MarrowCellDLD sorting of induced-OP9 adipocytes, we tested the device with spontaneously differentiated OP9 samples, where mature adipocytes are rare and need further enrichment. Even in this case, MarrowCellDLD consistently isolated the desired fraction with a reproducible separation cutoff achieving 90% purity. Our method was further tested with the MSOD human cell line, where we could retrieve intact MSOD BMAds at 94% purity. Going beyond bone marrow adipocytes, MarrowCellDLD was effective in sorting megakaryocytes, another notoriously difficult-to-isolate large and fragile cell type in the BM microenvironment. Cells above the separation cutoff were collected at 96% purity within the large cell fraction and showed characteristic MK polyploidy.

Considering a sample of 1.5 million cells, MarrowCellDLD showed a comparable processing time to FACS (Table 1). The ease of sample preparation without labelling, washing, or buffer exchange, contributed to a substantial reduction in the total processing time. Moreover, we operated the microfluidics at a relatively large flow rate (up to 1 mL/h) without compromising cell viability, and we believe that the flow speed could be even increased with acceptable post-sorting functionality. The design of MarrowCellDLD could also be adapted to have multiple chambers in parallel as an easy strategy to increase the throughput.

Overall, the current MarrowCellDLD system enables the recovery of at least 10^5^–10^6^ intact and functional fragile BM cells per sorting run, suitable for further studies and post-sorting culture. As a future perspective, we envision that the system could be adapted to sort primary murine and human samples and applied to isolate directly multiple differentiation stages from the original mixture. Processing samples from biopsies would require optimization of sample preparation pre-sorting and possibly adaptation of the MarrowCellDLD separation cutoff for human samples. The device could be used to isolate BMAds and megakaryocytes from the remaining blood cells (hematopoietic cells, stromal cells, and red blood cells) obtained from liquid bone marrow aspirates  as these two populations stand out with their larger size as compared to the remaining cell types in the bone marrow tissue. A potential scale-up use for clinical applications would also require a larger throughput that could be obtained by enlarging the channel height and by parallelization. The isolation of pure populations of large, fragile BM-derived cells (BMAds and MKs) with a precisely defined size range offers biologists new possibilities to study how each subpopulation interacts with neighboring cells regulating blood production and blood clotting. Studying the roles of these cells within the bone marrow niche can provide insights and reveal novel therapeutic targets for various hematological disorders, including leukemia, myeloproliferative neoplasms, and thrombocytopenia, as well as diseases affecting bone health.

## Methods

### Mouse-derived mesenchymal stem cell (OP9) culture and differentiation

OP9 cells (provided by T. Nakano, Kyoto University, Japan) were cultured and differentiated as described in Campos et al.^50^. Cells plated at 20,000 cells/cm^2^ were maintained in MEM-α with GlutaMaxTM (Gibco, Cat. No. 32561) supplemented with 10% FBS (Gibco, Cat. No. 10101), and 1% penicillin/streptomycin (Gibco, Cat. No. 15140) at 5% CO_2_ and 37°C. Cells were split using a trypsin-EDTA solution for 5 min when subconfluent (80%). A differentiation cocktail composed of culture medium along with dexamethasone (Sigma, Cat. No. D2915, 1 μM in ethanol), isobutyl-methylxanthine (Sigma, Cat. No. I7018, 0.5 mM in DMSO), and insulin (Sigma, Cat. No. I0516, 5 μg/mL) was used to perform the adipocytic differentiation for 6 days. For spontaneous adipocytic differentiation, OP9 cells were kept in culture for 18 days. Every 3–4 days, half of the medium was replaced with fresh culture medium**.**

### Human marrow stromal cell (MSOD) culture and differentiation

MSOD cells, an immortalized human bone marrow stromal line, were obtained from Ivan Martin’s laboratory in Basel, Switzerland^53^. MSOD cells were maintained in MEM-α with HEPES (Thermofisher, Cat. No. 15630-056, 10 mM). When subconfluent, cells were split as for OP9 cells. A differentiation cocktail, composed of culture medium along with dexamethasone, isobutyl-methylxanthine, insulin, and rosiglitazone (AdipoGen, Cat. No. CR1-3571, 15 μM), was used for adipocytic differentiation for 21 days. When cells were seeded, the differentiation cocktail was added at double concentration. On days 6 and 18, half of the medium was replaced with fresh culture medium. On day 12, half of the medium was removed and replaced with a differentiation cocktail.

### Mouse and human-derived mesenchymal cell staining

#### For flow cytometry sorting and imaging using ImageStream (Cytek Bioscience)

Prior to flow cytometric sorting, cells were stained with LipidTOX™ Deep Red neutral lipid stain (Invitrogen, Cat. No. H34477, supplied as 1000X for standard assays) for 30 min at 37°C and DAPI (Axonlab, Cat. No. A4099.005, 5 mg/mL) for 10 min at room temperature. The cells were subsequently detached by incubating for 5 min in 0.05% trypsin (Gibco, Cat. No. 25300054) at 37°C. The OP9 cells were gently resuspended in PBS 1X before being filtered with a 100 μm cell strainer in FACS tubes.

After DLD sorting, the different sorted fractions were stained for LipidTOX™ Deep Red neutral lipid stain (Invitrogen, Cat. No. H34477, supplied as 1000X for standard assays) for 30 min at 37°C and DAPI (Axonlab, Cat. No. A4099.005, 5 mg/mL) for 10 min at room temperature prior subjected to Imagestream. We were limited to acquiring with ImageStream 100 viable single cells per sample due to time and volume handling limitations (Supplementary Fig. S4C).

#### For fluorescence microscopy imaging

At different time points during the differentiation time course, plated cells were stained with live fluorescence dyes: BODIPY™ (boron-dipyrromethene, Invitrogen Cat. No. D3922,10 ng/mL), or LipidTOX™ Deep Red neutral lipid stain (Invitrogen, Cat. No. H34477, supplied as 1000X for standard assays) for 30 min at 37°C. Cells were incubated with the dyes in FluoroBrite phenol red-free DMEM medium (Gibco, Cat. No. A1896701) supplemented with 10% FBS and 1% penicillin–streptomycin for 30 min at 37°C in the dark, washed twice with warm PBS 1X and imaged in FluoroBrite medium using EVOS 5000 imaging system (ThermoFisher, Cat. No. AMF5000).

### Human-derived megakaryocyte differentiation from primary progenitors

Protocols and use of human blood products obtained from healthy blood donors at the Transfusion Center Inter-regional (Epalinges, Switzerland) were approved by the Cantonal Commission on Ethics in Human Research (CER-VD), Swiss Ethics Committees on research involving humans. After the signature of informed consent by all participants, human CD34+ cells were isolated from discarded buffy coats obtained upon blood unit preparation by magnetic-activated cell sorting (MACS). Briefly, the buffy coat collected at the blood transfusion center was diluted in an equivalent volume of 2 mM EDTA in PBS 1X. 15 mL Ficoll Plaque (GE, Cat. No. 17-1440-03) was added in a 50 mL tube. 30 mL of the blood mixture was then added delicately on top of Ficoll. Cells were then centrifuged for 30 min at 400*g* without brake and washed with 2 mM EDTA PBS 1X. Cells were treated with red blood cell lysis buffer (BioLegend) for 2 min and washed with PBS 1X. For CD34+ cell isolation, cells were subjected to staining according to manufacturer protocol (CD34 microbead kit: Microbeads Miltenyi, Cat. No. 130-100-453).

Isolated CD34+ cells were seeded at a density of 50,000 cells/mL in Expansion Medium for 7 days. Expansion medium was composed of StemSpan Serum Free Expansion Medium (Stemcell Technologies, Cat. No. 09650) supplemented with 20 ng/mL StemSpan Megakaryocyte Expansion Supplement (Stemcell Technologies, Cat. No. 02696), 20 ng/mL human plasma-derived low-density lipoprotein (hLDL) (Stemcell Technologies, Cat. No. 02698), 1 µM StemReginin1 (SR1) (Biogems, Cat. No. 122499) and 1% GibcoTM Penicillin–Streptomycin–Glutamine (PSG) (Thermofisher, Cat. No. 10378016). At day 7, cells were centrifuged and seeded at a density of 100,000 cells/mL in a differentiation cocktail for 7 days. The differentiation cocktail was composed of StemSpan Serum Free Expansion Medium supplemented with 20 ng/mL hLDL, 1 µM SR1, 0.5 µg/mL human recombinant Thrombopoietin (TPO) (StemCell Technologies, Cat. No. 78210), and 1% PSG (Thermofisher, Cat. No. 10378016). Successful Differentiation was confirmed by flow cytometry using CD41 (APC, clone HIP8, Biolegend Cat. No. 303710, Dilution 1:200) and CD42b (PE, clone HIP1, Biolegend, Cat. No. 303906, Dilution 1:200) surface detection. Briefly, 20 µL of the cells were diluted with up to 100 µL with PBS 1X. Cells were then stained with CD41 and CD42b for 20 min and subjected to flow cytometry (Accuri C6 PLUS, BD).

After DLD sorting, the different sorted fractions were stained for polyploidy (Hoechst 33258, Invitrogen, Cat. No. H3570). Briefly, cells were washed in PBS 1X and centrifuged for 5 min at 200*g*. Cells were resuspended in 100 µL PBS 1X with 1ug/mL Hoechst 33258. Cells were incubated for 15 min at room temperature. Cells were then washed as previously and resuspended in 50 µL of PBS 1X prior subjected to Imagestream (Cytek Bioscience).

### Sample preparation for MarrowCellDLD

The device operation was first assessed by sorting polystyrene beads of 15, 18, and 30 µm in diameter (Spherotec Inc.). Beads are suspended in  PBS 1X  supplemented with 1% (w/v) BSA (Sigma, Cat. No. A7906) to prevent aggregation and adhesion to the channel walls.

Both OP9 and MSOD BMAds, as well as megakaryocytes, were sorted by MarrowCellDLD following the same sample preparation protocol. Cells were washed, detached, and resuspended in culture media supplemented with 16% of Optiprep (StemCell, Cat. No. 07820) to ensure a single-cell suspension and filtered with a 150 μm cell stainer (PluriSelect, Cat. No. 43-50,150-01) for clusters removal. The optimal cell concentration for MarrowCellDLD sorting was 10^6^ cells/mL.

### Device design and fabrication

Our MarrowCellDLD sorting module is based on the design proposed originally by Huang et al*.*^35^*.* The microfluidic device has a single central inlet for sample delivery and two side ones for cell stream focusing. Two outlets collect two separated fractions. Microfluidic resistors were placed at the inlets to calibrate the applied pressure and at outlets for even splitting of the output flow. The DLD array was designed to provide a specific critical size for sorting according to the empirical model by Davis et al*.*^39^*.* We designed and fabricated devices at nominal critical sizes: 15, 17.5, 20, 22.5, and 25 μm. These five different arrays are designed by using a row shift factor equal to 15 and by varying the gap sizes: 36.7, 42.8, 48.9, 55, and 61.1 μm. Lateral and vertical gaps are equal. The nominal gap sizes were used for the SU-8 mold fabrication by photolithography, and the actual gap sizes obtained in the PDMS replicas were larger than the nominal ones. The nominal critical sizes of 15 µm and 20 μm were selected for further characterization and resulted in separation cutoffs of respectively 19 μm and 24 μm for induced-OP9 sorting.

The microfluidic device consisted of a polydimethylsiloxane (PDMS) module bonded to a glass substrate. The PDMS casting mold was fabricated by photolithography patterning of 50 μm SU-8 resist (Microchem 3025, Microresist Technologies, Berlin, Germany) on silicon. Surface conditioning of the silicon/SU-8 mold by silanization with Chlorotrimethylsilane (TMCS, Sigma, Cat. No. 386529) aided the PDMS release. After silanization, a mixture of PDMS precursor and crosslinker (SYLGARD™ 184 Silicone Elastomer Kit, Dow Corning Corp.) at a 1:9 ratio is dispensed onto the mold and cured by heating at 80°C for 2 h. Finally, the PDMS module is activated by oxygen plasma (550 mTorr, 29 W, for 45 s) and bonded onto a glass coverslip. The bonding is accelerated by a 2-min baking step at 80°C.

### MarrowCellDLD sorting

Vials were connected to inlets and outlets by Tygon^®^ tubing (Cole-Parmer, Cat. No. GZ-06420–02) paired with metallic connectors (Unimed, Cat. No. 200.010-A). Inlet reservoirs were paired with a pressure pump (Fluigent, Flow EZ, Cat. No. LuFEZ-1000) driving the flow. The chip was primed with cell culture media overnight before operation. After sample preparation, the mixture was delivered to the central inlet reservoir and kept under magnetic stirring to ensure homogeneity. The cellular samples were injected by applying a 20 mbar pressure to the central inlet reservoir, which corresponded in our geometry to a flow rate of approximately 8 μL/min. The sample stream was focused adjusting the two sheath flows by pressures in the range of 1–10 mbar. To avoid clogging issues throughout the whole sorting experiment (1–5 h), a continuous filtering method was developed by embedding a 100 μm cell strainer membrane at the tube extremity. The device is mounted on a microscope (Leica DM IL LED) equipped with a camera (ORCA-Flash4.0 V3 Digital CMOS camera) to visualize the cells trajectories in real time. After sorting, the two fractions were collected for further analysis.

### Statistical analysis

Results are displayed as Mean ± SD, unless otherwise stated, by Origin 2022 (OriginLab Corporation, Northampton, MA, USA). Two-tailed Student’s t-test for independent samples was used to test statistical significance, after Shapiro–Wilk normality test.

### Supplementary Information

Supplementary Figures.

Supplementary Video 1.

Supplementary Video 2.

## Data Availability

The datasets generated during and/or analyzed during the current study as been deposited in Zenodo (https://zenodo.org/records/8318213).
